# Supplementary material for: Prolonged preoperative wait time associated with elevated postoperative thirty-day mortality following intracranial tumor craniotomy in adult patients: A retrospective cohort study
Source: PLoS One. 2025 Jun 2;20(6):e0324928. doi: 10.1371/journal.pone.0324928 (PMC12129183; doi:10.1371/journal.pone.0324928)
Supplement: S1 Table — (DOCX) [file pone.0324928.s001.docx]

**S1 Table** Surgical site, tumor type and corresponding CPT codes

| CPT codes | Surgical procedures |
| --- | --- |
| 61510 | Supratentorial craniotomy for tumor |
| 61512 | Supratentorial craniotomy for meningioma |
| 61518 | Infratentorial craniotomy for tumor: others |
| 61519 | Infratentorial craniotomy for meningioma |
| 61520 | Infratentorial craniotomy for tumor: cerebellopontine angle |
| 61521 | Infratentorial craniotomy for tumor: midline |
| 61526 | Trans labyrinthine approach for cerebellopontine angle tumor |
| 61575 | Transoral approach to skull base, brain stem, or upper spinal cord for biopsy, decompression,or excision of lesion |
| 61545 | Craniotomy for craniopharyngioma |
| 61546 | Craniotomy for pituitary macroadenoma |
| Tumor type | CPT codes |
| Uncertain type tumor | 61510, 61518, 61521, 61575, others |
| Meningioma | 61512, 61519 |
| Cerebellopontine angle tumor | 61520, 61526 |
| Craniopharyngioma | 61545 |
| Pituitary macroadenoma | 61546 |
| Surgical site | CPT codes |
| Supratentorial | 61510, 61512 |
| Infratentorial or posterior fossa | 61518, 61519, 61520, 61521, 61526, 61575 |
| Sellar region | 61545, 61546 |
| Others | See details in “[https://doi.org/10.1371/journal.pone.0235273](https://doi.org/10.1371/journal.pone.0235273.s001)” |
