# Supplementary material for: Prolonged preoperative wait time associated with elevated postoperative thirty-day mortality following intracranial tumor craniotomy in adult patients: A retrospective cohort study
Source: PLoS One. 2025 Jun 2;20(6):e0324928. doi: 10.1371/journal.pone.0324928 (PMC12129183; doi:10.1371/journal.pone.0324928)
Supplement: S2 Table — (DOCX) [file pone.0324928.s002.docx]

**S2 Table** The univariate analyses of thirty-day mortality

|  | Statistics | OR (95%CI) | P-value |
| --- | --- | --- | --- |
| **Primary Exposure** |  |  |  |
| Wait time (Mean ± SD) | 1.39 ± 2.31 | 1.176 (1.142, 1.210) | <0.0001 |
| **Demographic characteristics** |  |  |  |
| Sex, N (%) |  |  |  |
| male | 8653 (47.29) | Ref |  |
| female | 9645 (52.71) | 0.627 (0.518, 0.759) | <0.0001 |
| Age ranges (years), N (%) |  |  |  |
| 18-40 | 2984 (16.31) | Ref |  |
| 41-60 | 7637 (41.74) | 2.747 (1.716, 4.398) | <0.0001 |
| 61-80 | 7084 (38.71) | 4.951 (3.129, 7.834) | <0.0001 |
| >80 | 593 (3.24) | 14.847 (8.817, 25.002) | <0.0001 |
| Race, N (%) |  |  |  |
| White | 13065 (71.40) | Ref |  |
| Black | 1217 (6.65) | 0.780 (0.508, 1.196) | 0.2538 |
| Asian | 526 (2.87) | 0.625 (0.308, 1.268) | 0.1927 |
| Native | 109 (0.60) | 1.146 (0.362, 3.629) | 0.8173 |
| Unknown | 3381 (18.48) | 1.145 (0.906, 1.447) | 0.2581 |
| Smoking status, N (%) |  |  |  |
| No | 14752 (80.62) | Ref |  |
| Yes | 3546 (19.38) | 1.127 (0.895, 1.420) | 0.3097 |
| **Preoperative Laboratory Indicators** |  |  |  |
| Na (Mean ± SD) | 138.63 ± 3.15 | 0.914 (0.889, 0.939) | <0.0001 |
| BUN (Mean ± SD) | 17.38 ± 7.90 | 1.043 (1.036, 1.051) | <0.0001 |
| WBC (Mean ± SD) | 9.50 ± 4.40 | 1.076 (1.059, 1.094) | <0.0001 |
| HCT (Mean ± SD) | 40.34 ± 4.73 | 0.926 (0.910, 0.942) | <0.0001 |
| INR (Mean ± SD) | 1.02 ± 0.20 | 1.399 (1.161, 1.687) | 0.0004 |
| **Preoperative Clinical Characteristics** |  |  |  |
| BMI (Mean ± SD) | 28.76 ± 6.61 | 0.988 (0.973, 1.003) | 0.1083 |
| Functional health status, N (%) |  |  |  |
| Independent | 17559 (95.96) | Ref |  |
| Partially Dependent | 648 (3.54) | 4.071 (3.019, 5.489) | <0.0001 |
| Totally Dependent | 91 (0.50) | 7.617 (4.197, 13.823) | <0.0001 |
| Ventilator dependent, N (%) |  |  |  |
| No | 18098 (98.91) | Ref |  |
| Yes | 200 (1.09) | 4.123 (2.516, 6.755) | <0.0001 |
| Steroid use for chronic condition, N (%) |  |  |  |
| No | 15571 (85.10) | Ref |  |
| Yes | 2727 (14.90) | 2.336 (1.917, 2.919) | <0.0001 |
| Preoperative blood transfusion, N (%) |  |  |  |
| No | 18241 (99.69) | Ref |  |
| Yes | 57 (0.31) | 4.804 (2.051, 11.255) | 0.0003 |
| ASA classification, N (%) |  |  |  |
| No/Mild Disturb | 5002 (27.34) | Ref |  |
| Severe Disturb | 10843 (59.26) | 4.064 (2.763, 5.977) | <0.0001 |
| Life Threat | 2416 (13.20) | 11.999 (8.052, 17.882) | <0.0001 |
| Moribund | 37 (0.20) | 20.786 (6.919, 62.443) | <0.0001 |
| **Preoperative Comorbidities** |  |  |  |
| Severe COPD, N (%) |  |  |  |
| No | 17490 (95.58) | Ref |  |
| Yes | 808 (4.42) | 2.278 (1.639, 3.167) | <0.0001 |
| Diabetes, N (%) |  |  |  |
| No | 16177 (88.41) | Ref |  |
| Yes(Insulin) | 778 (4.25) | 2.623 (1.895, 3.630) | <0.0001 |
| Yes(Oral) | 1343 (7.34) | 1.590 (1.163, 2.173) | 0.0036 |
| Hypertension, N (%) |  |  |  |
| No | 11325 (61.89) | Ref |  |
| Yes | 6973 (38.11) | 2.326 (1.921, 2.817) | <0.0001 |
| Congestive heart failure, N (%) |  |  |  |
| No | 18244 (99.70) | Ref |  |
| Yes | 54 (0.30) | 6.097 (2.7540, 13.567) | <0.0001 |
| Renal failure/Dialysis, N (%) |  |  |  |
| No | 18237 (99.67) | Ref |  |
| Yes | 61 (0.33) | 7.117 (3.485, 14.533) | <0.0001 |
| Disseminated cancer, N (%) |  |  |  |
| No | 14347 (78.41) | Ref |  |
| Yes | 3951 (21.59) | 2.880 (2.378, 3.488) | <0.0001 |
| Open wound infection, N (%) |  |  |  |
| No | 18148 (99.18) | Ref |  |
| Yes | 150 (0.82) | 5.330 (3.188, 8.911) | <0.0001 |
| Preoperative systemic infection, N (%) |  |  |  |
| No | 17653 (96.48) | Ref |  |
| SIRS | 602 (3.29) | 2.336 (1.606, 3.398) | <0.0001 |
| Sepsis/Septic Shock | 43 (0.23) | 13.037 (6.381, 26.634) | <0.0001 |
| Bleeding disorders, N (%) |  |  |  |
| No | 17936 (98.02) | Ref |  |
| Yes | 362 (1.98) | 2.161 (1.332, 3.505) | 0.0018 |
| **Intraoperative Characteristics** |  |  |  |
| Operation time (Mean ± SD) | 211.25 ± 131.65 | 0.998 (0.998, 0.999) | 0.0003 |
| Surgical site, N (%) |  |  |  |
| Supratentorial | 14295 (78.12) | Ref |  |
| Infratentorial or posterior fossa | 3681 (20.12) | 1.004 (0.794, 1.270) | 0.9726 |
| Sellar region | 244 (1.33) | 0.499 (0.159, 1.564) | 0.2328 |
| Others | 78 (0.43) | 0.520 (0.072, 3.746) | 0.5167 |
| Tumor type, N (%) |  |  |  |
| Uncertain type tumor | 12788 (69.89) | Ref |  |
| Meningioma | 4208 (23.00) | 0.392 (0.291, 0.529) | <0.0001 |
| Cerebellopontine angle tumor | 1058 (5.78) | 0.544 (0.333, 0.887) | 0.0148 |
| Craniopharyngioma | 98 (0.54) | 0.343 (0.048, 2.467) | 0.2879 |
| Pituitary macroadenoma | 146 (0.80) | 0.462 (0.114, 1.873) | 0.2798 |
| Emergency case, N (%) |  |  |  |
| No | 17127 (93.60) | Ref |  |
| Yes | 1171 (6.40) | 2.864 (2.202, 3.723) | <0.0001 |
| Wound classification, N (%) |  |  |  |
| Clean | 17788 (97.21) | Ref |  |
| Clean-Contaminated | 218 (1.19) | 0.567 (0.181, 1.779) | 0.3297 |
| Contaminated | 226 (1.24) | 0.733 (0.271, 1.978) | 0.5386 |
| Dirty/Infected | 66 (0.36) | 5.608 (2.661, 11.818) | <0.0001 |

BMI: Body-mass index; Na: Serum sodium; BUN: blood urea nitrogen; WBC: White blood cells; HCT: hematocrit; INR: International normalized ratio; COPD: chronic obstructive pulmonary disease; SD: standard deviation; OR, odds ratio; 95% CI, 95% confidence interval; Ref, reference.
