# Supplementary material for: Prolonged preoperative wait time associated with elevated postoperative thirty-day mortality following intracranial tumor craniotomy in adult patients: A retrospective cohort study
Source: PLoS One. 2025 Jun 2;20(6):e0324928. doi: 10.1371/journal.pone.0324928 (PMC12129183; doi:10.1371/journal.pone.0324928)
Supplement: S3 Table — (DOCX) [file pone.0324928.s003.docx]

**S3 Table** The comparison of multivariate analysis results from three datasets

| Datasets | Multiple imputation | Mean/Median imputation | Complete case |
| --- | --- | --- | --- |
|  | (N = 18,298) | (N = 18,298) | (N = 14,781) |
| Exposure | HR ( 95% CI ) P-value | HR ( 95% CI) P-value | HR ( 95% CI ) P-value |
| Wait time | 1.075 (1.040-1.110) 0.00001 | 1.075 (1.040, 1.110) 0.00001 | 1.072 (1.036-1.110) 0.00006 |
| Wait time group |  |  |  |
| <1 day | Ref | Ref | Ref |
| 1-7days | 1.519 (1.223, 1.886) 0.00015 | 1.513 (1.219, 1.880) 0.00018 | 1.459 (1.156, 1.840) 0.00144 |
| >7days | 2.072 (1.406, 3.052) 0.00023 | 2.065 (1.402, 3.041) 0.00024 | 2.014 (1.344, 3.017) 0.00069 |
| P for trend | <0.001 | <0.001 | <0.001 |

HR, hazard ratio; 95% CI, 95% confidence interval; Ref, reference.

The model fully adjusted for sex, age ranges, tumor type, functional status, ventilator dependent, COPD, diabetes, hypertension, CHF, renal failure/dialysis, disseminated cancer, steroid use, preoperative systemic infection, open wound infection, bleeding disorders, preoperative blood transfusion, emergency case, wound classification, ASA classification, Na, BUN, WBC, HCT, INR and Operation time.
