# Supplementary material for: Prolonged preoperative wait time associated with elevated postoperative thirty-day mortality following intracranial tumor craniotomy in adult patients: A retrospective cohort study
Source: PLoS One. 2025 Jun 2;20(6):e0324928. doi: 10.1371/journal.pone.0324928 (PMC12129183; doi:10.1371/journal.pone.0324928)
Supplement: Attachment — Submitted filename: review comment.docx [file pone.0324928.s005.docx]

**Thank you for the opportunity to review the manuscript regarding “Prolonged Preoperative Wait Time Associated with Elevated Postoperative Thirty-Day Mortality Following Intracranial Tumor Craniotomy in Adult Patients: A Retrospective Cohort Study”. The manuscript provided the vital information for preoperative issue with large sample size. However, there are some issue to be clarify for the improvement and reproducibility.**

Introduction

The introduction was quite long (5 paragraphs). Please rewrite the concise introduction and the clear point of the rationale of the study.

Methodology

1. The methodology section was quite brief considering the possibility of reproducibility but the statistical analysis was too long, complex and sophisticate for normal reader (non-statistician) to understand. The author should focus on the detail of the variable selection.

- Since the secondary data came from different institute, what was the definition of each variable? The separate section of the main exposure (the definition, how to determine), outcome of the study (defined the time to event and censored), and potential confounding variables dividing into preop-, intraop-, and postoperative factor should be provided.

- Perioperative factor and complication may affect the outcome of 30 day-mortality after craniotomy. The authors should provide intraoperative adverse event (intraoperative hypotension, hypercarbia, metabolic acidosis), the intraoperative factors (estimated blood loss, blood transfusion, crystalloid volume), and postoperative complications such as bleeding disorder, intracranial hypertension, brain edema, or repeated surgery, that can lead to deteriorate condition postoperatively.

2. Please summary to the statistical analysis section to be simple, concise for non-statistician to understand and describe how to select confounding variable for propensity matching since it is important to include intraoperative and postoperative complication as well.

3. Sample size determination was missing in the manuscript. Considering the large sample size, the small differences can lead to statistically significant difference but not clinically significant. Therefore, it is better to present the proper sample size based on the primary objective.

Results

1. For Cox regression analysis, the cumulative hazard survival curve or Kaplan-Meier curve of 30- day mortality among short and long wait times should be provide to simpler visualize the risk/rate of the 30-day mortality among short and long wait times.
2. Variables in Table 1 was quite confusing. Which variables were postoperative factor (bleeding disorder??), the authors should differentiate the covariates into preop-, intraop-, and postoperative factor since they could be the important factors that lead to mortality after surgery.

Discussion

Please provide the implication of the study in the discussion, so the reader/physician can apply for clinical practice in their hospital setting.
